# Leaf anatomy of a late Palaeozoic cycad

**DOI:** 10.1098/rsbl.2017.0456

**Published:** 2017-11-01

**Authors:** Zhuo Feng, Yong Lv, Yun Guo, Hai-Bo Wei, Hans Kerp

**Affiliations:** 1 Institute of Deep Time Terrestrial Ecology, Yunnan University, Kunming 650091, People's Republic of China; 2 Research Center for Earth System Science, Yunnan University, Kunming 650500, People's Republic of China; 3 State Key Laboratory of Palaeobiology and Stratigraphy, Nanjing Institute of Geology and Palaeontology, Chinese Academy of Sciences, Nanjing 210008, People's Republic of China; 4 Key Laboratory of Economic Stratigraphy and Palaeogeography, Nanjing Institute of Geology and Palaeontology, Chinese Academy of Sciences, Nanjing 210008, People's Republic of China; 5 Institute of Karst Geology, Chinese Academy of Geological Sciences, Guilin 541004, People's Republic of China; 6 Forschungsstelle für Paläobotanik, Westfälische Wilhelms-Universität, Heisenbergstrasse 2, 48149 Münster, Germany

**Keywords:** *Plagiozamites*, cuticle, cycads, late Palaeozoic, China

## Abstract

Today, cycads are a small group of gymnospermous plants with a limited distribution in the (sub)tropics, but they were major constituents of Mesozoic floras. Fossil leaves sporadically found in latest Carboniferous and Permian floras have putatively been ascribed to cycads. However, their true affinity remains unclear due to the lack of anatomical evidence. Virtually all modern cycads have pinnate leaves, but this type of leaf morphology is by no means unique for cycads. Pinnate leaves of *Plagiozamites oblongifolius* Halle 1927 with well-preserved cuticles showing the epidermal anatomy are here described from the upper Permian Xuanwei Formation of Yunnan Province, Southwest China. The cuticles show a clear differentiation into costal and intercostal zones; stomata are confined to the intercostal zones on both the upper and lower leaf surfaces. The external morphology and the epidermal anatomy of these fossil leaves are closely comparable with those of extant cycads, particularly members of the family Zamiaceae.

## 1. Background

Cycads are gymnosperms, typically characterized by a usually unbranched woody trunk with an green crown of pinnate, stiff leaves. Individual plants are either male or female [1]. The Order Cycadales comprises three extant families with approximately 300 species accommodated into 10–12 genera [2]. A recent phylogenetic analysis of extant species suggests that most living cycads evolved from a common ancestor that lived around 12 Ma (late Miocene) [3,4]. Despite being minor constituents of (sub)tropical floras today, during the Mesozoic, cycads were extremely common according to the rich and diverse fossil record [5].

Some leaves and reproductive organs from the upper Palaeozoic have been putatively assigned to cycads. Owing to their unique morphology, the attribution of reproductive organs to cycads is usually undisputed, although it should be noted that some fertile remains originally attributed to cycads turned out to be of pteridospermous rather than of cycadalean affinity [6]. Much more problematic is the nature of late Palaeozoic cycad-like foliage, which although still rare, is far more common than reproductive organs. Pinnate leaves are not unique for cycads and an assignment of sterile foliage to cycads is often only possible on the basis of cuticles. In particular, bennettitaleans, a group of Mesozoic gymnosperms of which the origins are still enigmatic, had leaves that are very difficult to distinguish from those of cycadaleans on the basis of macroscopic characters. Also the late Palaeozoic noeggerathialeans had very similar pinnate leaves. Unfortunately, only few, very poorly preserved cuticles of late Palaeozoic cycad-like leaves have been described so far. Herein, we document leaves of *Plagiozamites oblongifolius* Halle 1927 from the upper Permian of Southwest China with well-preserved cuticles showing a combination of features typical for cycadaleans, thus providing a new insight into the early cycads.

## 2. Material and methods

Fossil leaves with cuticle were collected from the lower member of the Xuanwei Formation in the Damogou, Huangjiaochong and Qingyun coalmines of Housuo Town, Fuyuan County, Yunnan Province, China. The lower and upper members of the Xuanwei Formation are, respectively, Wuchiapingian and Changhsingian in age [7].

Plant leaf remains were picked from the rock surface, and macerated using Schulze's reagent (30% HNO_3_ with a few crystals of KClO_3_) followed by treatment with 5% KOH [8]. Macerated cuticles were washed in distilled water, dehydrated in pure glycerine and subsequently mounted in permanent glycerine-jelly slides. The obtained cuticles were studied by using a Leica DM 2500 M transmitted-light microscope (equipped with fluorescence) and an FEI QUNTA field emission gun 650 scanning electron microscope (SEM).

## 3. Results

**Figure 1.**
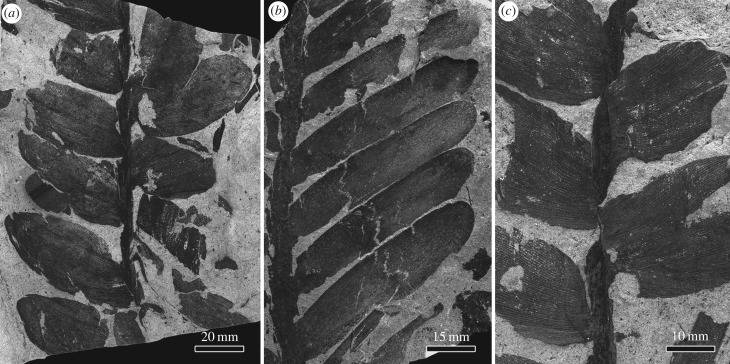
Fossil cycad from the upper Permian of Southwest China. (*a–c*) Photographs of the pinnate leaf of *P. oblongifolius*; note the plagiotropically attached leaflets along the thick rachis.

**Figure 2.**
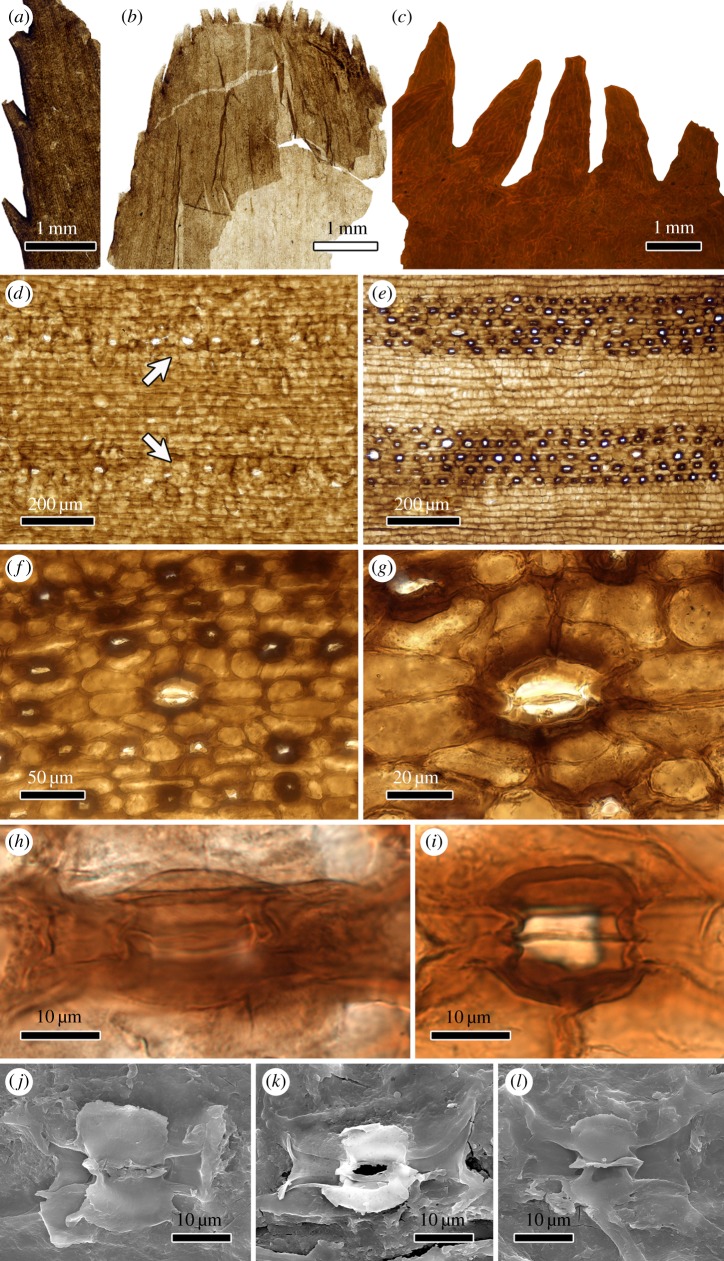
Cuticle of *P. oblongifolius* from the upper Permian of Southwest China. (*a*) The abaxial cuticle of the distal leaflet showing each vein corresponding to a fine tooth. (*b*) The rounded apex with fine teeth. (*c*) Fluorescence image showing the detailed structure of the fine teeth on the distal leaflet apex. (*d*) The adaxial cuticle showing less common occurrence of stomata (arrows). (*e*) The abaxial cuticle. Note the stomatal bands confined to the intercostal zones. (*f*) The abaxial cuticle, showing a larger stoma occurring among the smaller stomata. (*g*) Close-up of *f*, showing a larger stoma incorporated with eight subsidiary cells. (*h*,*i*) Light-microscopic images showing the smaller stomata on the abaxial cuticle. (*j–l*) SEM images showing the smaller stomata on the abaxial cuticle; note the kidney-shaped guard cells of the stomatal complex, overarched by four to six subsidiary cells.

Cycad-like leaves commonly occur in the Xuanwei Formation. The leaf shows two rows of partially overlapping ovate to oblong leaflets, obliquely attached to the axis (figure 1*a–c*). The semi-clasping leaflets are coriaceous, with a number of veins extending from the leaflet base, which bifurcate and diverge. Each vein ends in a fine tooth at the margin or the apex of the leaflets (figure 2*a–c*).

Cuticles show well-developed costal and intercostal zones, both on the adaxial and abaxial cuticles (figure 2*d*,*e*). The epidermal cells overlying the veins are elongate, rectangular, regularly arranged with their long axes parallel to the vein; cells of the intercostal zones range from elongate rectangular to polygonal or broadly rectangular. Papillae and trichomes are absent. The deeply sunken stomata are present on both leaf sides (amphistomatic), but exclusively confined to the intercostal zones; stomatal apertures are oriented more or less parallel to the veins in both the adaxial and abaxial cuticle. The interior parts of the subsidiary cells bounding the stomatal pore are much more strongly cutinized than the exterior parts, often forming a prominent ring-like structure encircling the stomatal pore (figure 2*e*,*f*). In the adaxial cuticle, stomatal complexes are less common and much larger in diameter than in the abaxial cuticle. Stomata occur in the central region of the intercostal zones, either individually or aligned in rows (figure 2*d*; arrows). In the abaxial cuticle, stomata are arranged in bands between adjacent veins (figure 2*e*). Few larger stomatal complexes (figure 2*f*,*g*) consisting of six to eight subsidiary cells occur irregularly among the smaller stomata. The smaller stomatal complexes comprise four to six subsidiary cells and slightly vary in shape and size. The two kidney-shaped guard cells are thinly cutinized and overarched by the subsidiary cells (figure 2*h*,*i*). When preserved, they are only clearly visible by SEM observations (figure 2*j–l*).

## 4. Discussion

The fossil leaves described here are characterized as pinnate fronds with obliquely attached leaflets with multiple, more or less parallel veins and deeply sunken haplocheilic stomata, a combination of features typical for fossil and extant Cycadales [1,9]. The individual leaflets within a single leaf may differ in size and shape, in the angle of insertion and the number of veins, depending on the position in and the size of the leaf. This is also known from extant cycads [9]. The distinctive combination of macro- and micromorphological features excludes a possible affinity of the current leaves to late Palaeozoic seed ferns, ginkgophytes and conifers.

Owing to their unique morphology, megasporophylls provide the strongest evidence for the occurrence of true cycads during the late Palaeozoic. *Dioonitocarpidium* sp. from the early Permian lower Pease River Group of the USA has a pinnate distal blade and basal part with seeds attached. It is considered to be the oldest known *Cycas*-like megasporophyll [10]. More diverse cycadalean reproductive organs have been documented from the middle and upper Permian of China. *Primocycas chinensis* Zhu and Du 1981 from the middle Permian Lower Shihhotse Formation of North China is assigned to the cycads based on its morphological resemblance to modern *Cycas* megasporophylls [11]. Several other middle Permian cycad-like megasporophylls from North China have been assigned to the genus *Crossozamia* Pomel 1894 [12]. Microsporophyll fossils of probable cycadalean affinity from the middle and upper Permian of North China were described as *Liulinia lacinulata* Wang 1986, *Cycadostrobilus paleozoicus* Zhu 1994 and *Pania cycadina* Yang 2006 [13–15]. Unfortunately, no cuticle has been preserved from any of these organs.

Only very few cuticles of late Palaeozoic cycad-like foliage are known. These include a few inconclusive fragments without stomata illustrated by Zeiller [16]. Barthel [17] figured some dispersed cuticles from the Rotliegend of eastern Germany clearly showing a cycadalean epidermal anatomy; this material was obtained from bulk macerations and there is no information on the leaf morphology. More recently, Pott *et al*. [18] presented a few fragmentary cuticles of *Pterophyllum samchokense* Kawasaki 1934 from the Permian of North China. Although these cuticle fragments are too small to show the arrangement of stomata and the preservation is very poor, the shape of the stomatal complexes may suggest a cycadalean affinity; the species was transferred from *Pterophyllum* Brongniart 1825, a genus emended to include only bennettitalean foliage, to *Pseudoctenis* Seward 1911 [18].

The material described here is a Palaeozoic cycad-like leaf with well-preserved cuticles showing the arrangement of epidermal cells and stomata, and the structure of the stomatal complex. These features indicate a cycadalean affinity. Based on the gross morphology, our specimens can be identified as *P. oblongifolius* Halle 1927. *Plagiozamites* Zeiller 1894 is a genus for fossil foliage known from the uppermost Carboniferous and Permian of Europe and China. The systematic affinity of *Plagiozamites* has long been a matter of debate. Zeiller [19] compared *Plagiozamites* to *Zamites* Brongniart 1828, a Mesozoic taxon now classified as a bennettitalean, whereas others consider it to be a Noeggerathialean [5].

Guo *et al*. [20] illustrated a cross-section through an anatomically preserved rachis that was identified as *P. oblongifolius*. This axis was originally described as having a U-shaped vascular bundle similar to that of the modern *Cycas*. Wang *et al*. [21] refigured this specimen and characterized the vascular bundle as Ω-shaped. Based on the close association with noeggerathialean cones, they assigned this rachis to the Noeggerathiales, an enigmatic group of Progymnosperms. However, this type of rachis was never found in organic connection with undisputed noeggerathialean remains.

The cuticle of *P. oblongifolius*, having many parallel costal and intercostal zones, and stomatal complexes, each with a ring of four to eight subsidiary cells, is in all respects, very similar to that of the modern and Mesozoic–Cenozoic cycads and based on the cuticle can easily be distinguished from other groups having cycad-like foliage. *Tingia* Halle 1925, a superficially similar type of foliage assigned to the noeggerathialeans [22], has cuticles that differ in having simple stomata, without well-developed subsidiary cells, that are aligned in grooves parallel to the veins [23]. Bennettitaleans can easily be distinguished by their syndetocheilic stomata [5]. A pteridospermous nature of *Plagiozamites* is highly unlikely because of the gross morphology of the leaves; there are no pteridosperms with pinnate leaves bearing such leaflets. The stomatal architecture of *P. oblongifolius* resembles that of some extant *Macrozamia* and *Encephalartos* species, especially the prominent ring-shaped thickenings on the interior side of the subsidiary cells. *Macrozamia* Miquel 1842 and *Encephalartos* Lehmann 1834 both belong to the family Zamiaceae, a more advanced clade of cycadaleans [1].

Two size classes of the stomatal complexes are found in the adaxial cuticle of the *P. oblongifolius* leaflets. The large-sized stomata are very rare and irregularly present among the small-sized stomata, but no conspicuous distribution pattern can be recognized. The larger stomata generally have more subsidiary cells (six to eight) than those of the smaller stomata (four to six), although the basic architecture of the large and small stomata is identical. The anatomical investigation of modern cycads leaves reveals that the size, arrangement and even the architecture of stomata may vary significantly in a single species [9,24]. Our material may indicate that these traits of the extant cycads also occurred in their ancestral forms that can be traced back to the late Palaeozoic time.

The recognition of *P. oblongifolius* as a true cycad supports a late Palaeozoic occurrence of cycads. Future research should concentrate on the cuticle studies of other types of possible late Palaeozoic cycadalean foliage and on their correlation with reproductive organs in order to reconstruct whole-plant taxa that will form a sound basis for unravelling the phylogeny of this intriguing group of primeval gymnosperms.

## Data accessibility

No datasets were generated or analysed during the current study. The specimen, slides and digital images are housed in the Palaeobotanical Collections of the Institute of Deep Time Terrestrial Ecology, Yunnan University, China, with catalogue numbers: YNUPB10001–YNUPB10003.
